# The impact of CwlM depletion on the susceptibility of *Mycobacterium smegmatis* to anti-tuberculosis drugs

**DOI:** 10.1371/journal.pone.0334937

**Published:** 2025-10-24

**Authors:** Shufeng Yang, Yuzhao Ren, Yu Wu, Xinyang Li, Xin Liu, Guoying Deng

**Affiliations:** Department of Pathogen Biology and Microecology, College of Basic Medical Sciences, Dalian Medical University, Dalian, China; Bennett University, INDIA

## Abstract

CwlM, identified as an N-acetylmuramoyl-l-alanine amidase, plays crucial roles in the synthesis and remodeling of peptidoglycan in mycobacteria. This protein also appears to participate in both drug susceptibility and tolerance mechanisms within these organisms. In our study, we employed CRISPR interference (CRISPRi) to deplete CwlM in *Mycobacterium smegmatis* (*M. smegmatis*) and examined the resulting effects on the susceptibility of mycobacteria to first-line anti-tuberculosis drugs, including isoniazid (INH), rifampicin (RIF), pyrazinamide (PZA), and ethambutol (EMB), as well as the β-lactams cefoxitin and imipenem. Our findings revealed that CwlM depletion increased the susceptibility of the bacterium to RIF, EMB, cefoxitin, and imipenem, while tolerance was heightened against INH and PZA. The enhanced antibiotic susceptibility can primarily be attributed to increased permeability of the bacterial cell wall. Conversely, the observed tolerance to INH might be ascribed to elevated expression of the amidase known as hydrazidase along with its LuxR-type regulator. Furthermore, several genes associated with peptidoglycan synthesis appeared to correlate with increased expression levels of either hydrazidase or its LuxR-type regulator. Collectively, these findings indicate that CwlM depletion significantly influences the susceptibility of *M. smegmatis* towards certain anti-tuberculosis drugs and may be implicated in drug susceptibility and tolerance mechanisms in *M. smegmatis*.

## Introduction

Infectious diseases caused by mycobacteria, especially *Mycobacterium tuberculosis* (*M. tuberculosis*), threaten human health worldwide. *M. tuberculosis* infects approximately one-quarter of the global population, being responsible for an estimated 1.0–1.5 million deaths annually [1].

Peptidoglycan (PG) is a crucial component of the mycobacterial cell wall. PG hydrolases play significant roles in cell division, peptidoglycan maturation, and fragment recycling [2,3]. Among these hydrolases, CwlM has been identified as an N-acetylmuramoyl-l-alanine amidase that cleaves N-acetylmuramoyl l-alanyl-d-isoglutamine, resulting in the release of free N-acetylmuramic acid [4]. Consequently, CwlM protein is involved in cell wall remodeling, and its gene is essential for the in vitro growth of *M*. *tuberculosis* and *M. smegmatis* [5,6]. Research has revealed the evolutionary biological characteristics of CwlM. Its phosphorylated form enhances the activity of MurA, the initial enzyme in the biosynthesis pathway of PG precursors, thereby promoting PG synthesis. However, CwlM is dephosphorylated during starvation, resulting in reduced MurA activity and diminished cell wall metabolism but increased tolerance to multiple antibiotics [5]. Thus, CwlM appears to be implicated in drug susceptibility and tolerance mechanisms within mycobacteria. In *M. abscessus*, CRISPR-mediated suppression of CwlM resulted in increased susceptibility to β-lactams, indicating complex interplay between genetic factors and pharmacological agents [7].

Isoniazid (INH), rifampicin (RIF), pyrazinamide (PZA), and ethambutol (EMB) constitute the first line of anti-tuberculosis medications [8,9]. β-lactam antibiotics, which target PG synthesis, represent a potential class of anti-tuberculosis agents [10]. To investigate the role of CwlM in drug susceptibility, we employed CRISPR interference (CRISPRi) to deplete CwlM in *M. smegmatis*, a commonly used surrogate model for pathogenic *M. tuberculosis*. We explored whether CwlM depletion affected mycobacterial susceptibility to first-line anti-tuberculosis drugs including INH, RIF, PZA, EMB, and β-lactam antibiotics such as cefoxitin and imipenem. Our findings indicated that CwlM depletion increased the susceptibility of mycobacteria to RIF, EMB, cefoxitin and imipenem, while enhancing its tolerance to INH and PZA. The enhanced susceptibility to antibiotics can be primarily attributed to improved permeability, whereas the observed tolerance to INH might be linked to elevated expression of a newly identified amidase known as hydrazidase [11].

## Materials and methods

### Bacterial strains and growth condition

*M. smegmatis* mc^2^155 was cultured in Middlebrook 7H9 broth (BD Biosciences, Franklin Lakes, NJ, USA) supplemented with 10% albumin–dextrose–catalase (ADC) or on Middlebrook 7H10 agar (BD Biosciences) enriched with 10% oleic–albumin–dextrose–catalase at 37°C. *Escherichia coli* was cultivated in Luria–Bertani broth. Kanamycin was added to the cultures at 25 µg/mL to facilitate the amplification of the CRISPRi backbone pLJR962 (Addgene #115162). Hygromycin at a concentration of 25 µg/mL was included for selecting for PzaA knockout strain. Stock solutions of INH, RIF, EMB, cefoxitin, and imipenem were prepared using ultrapure water at a concentration of 1 mg/mL. PZA is stored at a concentration of 10 mg/mL. Anhydrotetracycline (ATc, Sigma-Aldrich, St. Louis, MO, USA) was dissolved in dimethyl sulfoxide (Sigma-Aldrich) to achieve a stock concentration of 10 mg/mL.

### Construction of the CwlM-depleted strain

The CwlM-depleted strain was constructed using the CRISPRi approach in accordance with mycobacterial protocols [12]. Briefly, an sgRNA sequence (5′-GGGAAGTACCCGTCGACCAGGCCGGT-3′) was designed to target *CwlM* (MSMEG_6935). The forward and reverse oligonucleotides of the sgRNA were annealed and ligated to *Bsm*BI-digested pLJR962 to generate pLJR962-sgRNA. The pLJR962 plasmid (Addgene #115162) contains a dCas9 cassette and is specifically designed for use in *M. smegmatis*. The plasmid can express Sth1 dCas9 and the sgRNA from a Tet repressor-regulated promoter. Transcription from the TetR-regulated promoters is activated in the presence of ATc or doxycycline. The pLJR962-sgRNA plasmid was transformed into *E. coli* NovaBlue, and plasmid extraction was performed using Takara MiniBEST Plasmid Purification Kit (Takara, Dalian, China). The desired PLJR962-sgRNA plasmids were confirmed by Sanger sequencing. The recombinant plasmid was then electroporated into *M. smegmatis*. Colonies grown on Middlebrook 7H10 agar plates containing 25 µg/mL kanamycin were transferred to 7H9 broth with ADC and 0.05% Tween 80. Different concentrations of ATc (50, 100, and 200 ng/mL) were added to each culture. Growth for at least three generations permitted dCas9:sgRNA-mediated transcriptional interference.

### Assessment of CwlM depletion by quantitative reverse transcription real-time PCR (qRT-PCR)

Bacterial cultures, with or without ATc, were harvested via centrifugation at 3000 × *g* for 5 min. The resulting pellets were suspended and disrupted using ultrasonication. Chloroform was then added to the suspension, followed by centrifugation at 16,000 × *g* for 15 min. The supernatant was transferred to a separate tube. Sodium acetate and isopropanol were introduced to facilitate RNA precipitation. After washing with ethanol, the RNA was dissolved in RNase-free H_2_O. The quantity and purity of the isolated RNA were assessed using a Nanodrop 2000 spectrophotometer (Thermo Fisher Scientific, Waltham, MA, USA). qRT-PCR was conducted according to the instructions of the PrimeScript™ RT Reagent Kit (Takara) and TB Green® Premix EX Taq™ II (Tli RNaseH plus, Takara). The mean of CwlM expression was normalized by the housekeeping gene SigA. Three biological replicates, with each consisting of three technical replicates, were utilized for the quantification of the target sequences. The primers utilized in this study are listed in Table 1.

**Table 1:** Primers used in this study.

Primers	Primer sequences (5’-3’)
For quantitative reverse transcription PCR	
SigA F	GTGTGGGACGAGGAAGAGTC
SigA R	ACCTCTTCTTCGGCGTTGAG
CwlM F	TCATCCAGCGTGAAGTGGTG
CwlM R	GTCACCGGGATTGGTGATGT
PzaA F	GGTCAACTACTACACCGACC
PzaA R	GATCTCGACCTCGACAAGTT
KatG F	ACGAACTTCCCGATCACACC
KatG R	GGTCTGAGCTTCTCCGATGG
PncA F	TGAGCACTTCTCGGACACAC
PncA R	GCATCGAACTCCGGATGGAA
LuxR-type regulator F	GTGCCAAGGGTTTCAACCTG
LuxR-type regulator R	CACCGCTGTAGGAGTCGTAG
For DNA amplification	
KatG F	TTGCCTGAGGATCGCCCGATCG
KatG R	TCAGGCGACGTCGAAGCGGTC
PncA F	ATGCGTGCACTGATTGTCG
PncA R	TCAGCTGATCGTCACTCCGG
ΔPzaA F	CCACTAGTCACCCGAATCTGCAG
ΔPzaA R	GCCCACCTTCTCCAGAACC
Fragment of PzaA-HygR F	GCGCAGGGTCGCGGGCGCGAC
Fragment of PzaA-HygR R	GCACCCGCAGCACCGGCGGC
For DNA sequencing	
pJET F	CGACTCACTATAGGGAGAGCGGC
pJET R	AAGAACATCGATTTTCCATGGCAG

### Growth curve analysis

Cultures were grown to the logarithmic phase in 7H9 broth supplemented with ADC and Tween 80. Following dilution to OD_600_ of 0.05 in medium, cultures were induced with or without 100 ng/mL ATc and incubated at 37°C with shaking at 120 rpm. OD_600_ was measured every 12 h using a microplate reader (BioTek Instruments Inc., Winooski, VT, USA) until the stationary phase was reached. The experiments were performed in triplicates.

### Effect of CwlM depletion on *M. smegmatis* morphology

Cultures of *M. smegmatis*, both treated and untreated with ATc, were harvested by centrifugation before being fixed in a solution containing 3% glutaraldehyde. After fixation, samples were further processed in a solution containing 1% osmium tetroxide, followed by dehydration through a graded ethanol series (30%, 50%, 70%, 80%, 100%). Cellular morphology was examined utilizing scanning electron microscopy (SEM, JSM-840, JEOL, Tokyo, Japan).

### Antibiotic susceptibility assay

The minimal inhibitory concentrations (MICs), defined as the lowest drug concentration that inhibited more than 90% of bacterial growth, of INH, RIF, PZA, EMB, cefoxitin, and imipenem were determined using the broth microdilution technique. *M. smegmatis* cultures were grown to the logarithmic phase and then diluted to OD_600_ of 0.05. The cultures were divided into two equal volumes and incubated with or without the addition of 100 ng/mL ATc until they reached the logarithmic phase at OD_600_ of 0.4. A total of 1 × 10^7^ bacteria were introduced into the respective wells. The antibiotics were serially diluted at a 1:2 ratio and added to the wells, resulting in a total volume of 100µl per well. The final concentration ranges of the antibiotics were as follows: INH, 2–32 µg/mL; RIF, 0.25–4 µg/mL; PZA, 50–400 µg/mL; EMB, 0.125–2 µg/mL; cefoxitin, 2–32 µg/mL; and imipenem, 0.5–8 µg/mL. The plates were incubated for 3 days at 37°C. To assess cell viability, 10 μL of Alamar Blue solution was added to each well. The plates sealed in bags were further incubated for an additional 24 h at 37°C. The MIC value is defined as the lowest concentration of compound that inhibits growth as measured by inhibition of blue to pink conversion [12]. Simultaneously, cultures were serially diluted, followed by plating on 7H10 agar plates to count colonies.

### Ethidium bromide (EtBr) uptake assay

Cultures were cultivated with or without ATc until reaching OD_600_ of approximately 0.8 and then washed with phosphate-buffered saline (PBS, 137 mM NaCl, 2.7 mM KCl, 10 mM Na_2_HPO_4_, and 2 mM KH_2_PO_4_). Then, 200 μL from each dilution were transferred to black opaque flat-bottomed 96-well plates, in which bacterial cells were treated with 0.5 µg/mL EtBr. PBS served as a blank control. Dye accumulation was measured kinetically using excitation at 530 nm and emission detection at 590 nm and recorded every two minutes for 60 min in triplicate using a spectrophotometer (Varioskan LUX, Thermo Fisher Scientific).

### DNA sequencing of KatG and PncA

The genes encoding KatG and PncA were amplified by PCR utilizing the genome of the CwlM-depleted strain as a template. The resulting products were ligated into the pJET vector, generating the pJET-KatG and pJET-PncA plasmids, respectively. These constructs were subsequently transformed into NovaBlue *E. coli* strains for propagation. Following plasmid extraction, DNA sequencing was performed, and an alignment was conducted using wild-type sequences available on the Mycobrowser website. The primers utilized for DNA amplification and sequencing are detailed in Table 1.

### Transcription analysis of KatG, PncA, PzaA and LuxR-type regulator via qRT-PCR

To explore the potential mechanism of INH and PZA tolerance, qRT-PCR was performed to detect the transcription levels of KatG, PncA, PzaA and LuxR-type regulator. This analysis was performed following the instructions provided with PrimeScript™ RT Reagent Kit and TB Green® Premix EX Taq™ II. Three biological replicates, each counting three technical replicates, were performed. Primers utilized during this process were listed in Table 1.

### Purification, identification and quantification of PzaA protein

Purification of PzaA protein was performed as reported by Sakiyama A, et al [11]. *M. smegmatis* cells were harvested by centrifugation at10,000 × g for 10 min and washed with 50 mM sodium phosphate buffer (pH 7.0). After sonication, insoluble material was removed by centrifugation at 6,000 × g for 30 min. The PzaA protein was purified at 0−4°C through fractionation with 40−60% saturated ammonium sulfate, followed by dialysis. The fraction was applied to a HiTrap Q HP column (GE Healthcare, Chicago, IL, USA) and eluted with a 0–0.5 M NaCl gradient. Active fractions were mixed with 80% saturated ammonium sulfate to 20% saturation and loaded onto a HiTrap Butyl HP column (GE Healthcare), eluted with a 20−0% saturated ammonium sulfate gradient. The pooled active fractions were concentrated and applied to a Superdex 200 Increase column with 150 mM NaCl, followed by a Resource Q column (GE Healthcare), eluted with a 0–0.5 M NaCl gradient. The equal volumes of collected protein were subjected to purity check by SDS-PAGE and protein concentration measurement using a Bio-Rad Protein Assay Dye Reagent Concentrate (Bio-Rad, Hercules, CA, USA) with BSA as the standard. The band was excised from the gel for a further identification using mass spectrometry (MS).

### Construction of PzaA knockout strain by homologous recombination

The PzaA gene knock out strain, ΔPzaA, was constructed using homologous DNA recombination method in this study. The PzaA gene with its promoter was amplified using primers ΔPzaA-F and ΔPzaA-R (Table 1) from *M. smegmatis* mc^2^ 155 genome by *Pfu* DNA polymerase, and then was cloned to pJET 1.2/blunt vector to generate pJET-PzaA. pJET-PzaA was digested and ligated with HygR cassette to generate pJET-PzaA-HygR. After pJET-PzaA-HygR was digested by SpeI and NotI enzymes, the fragment of PzaA-HygR was purified and cloned to pPR27-xylE. pPR27-xylE-PzaA-HygR was electroporated to induced and uninduced strains, and the transformants were selected. PzaA knock out strains, ΔPzaA and ΔPzaA/CwlM-depleted strains, were confirmed by PCR. The primers used here were listed in Table 1.

### Transcriptomics analysis

Total RNA was isolated using the Trizol Reagent (Invitrogen Life Technologies). Quality and integrity were determined using a NanoDrop spectrophotometer (Thermo Scientific) and a Bioanalyzer 2100 system (Agilent). Zymo-Seq RiboFree Total RNA Library Kit was used to remove rRNA from total RNA. Random oligonucleotides and SuperScript III were used to synthesize the first strand cDNA. Then use RNaseH to degrade the RNA strand in DNA-RNA hybrid, and in the DNA polymerase I system, use dNTP with dUTP instead of dTTP as raw material to synthesize the second strand of cDNA. Remaining overhangs were converted into blunt ends via exonuclease/polymerase activities and the enzymes were removed. After adenylation of the 3′ ends of the DNA fragments, Illumina PE adapter oligonucleotides were ligated to prepare for hybridization. To select cDNA fragments of the preferred 400–500 bp in length, the library fragments were purified using the AMPure XP system (Beckman Coulter, Beverly, CA, USA). DNA fragments with ligated adaptor molecules on both ends were selectively enriched using Illumina PCR Primer Cocktail in a 15 cycle PCR reaction. Products were purified (AMPure XP system) and quantified using the Agilent high sensitivity DNA assay on a Bioanalyzer 2100 system (Agilent). The sequencing library was then sequenced on NovaSeq 6000 platform (Illumina) Shanghai Bioprofile Technology Company Ltd. The experiments were performed in triplicates.

### Statistical analysis

Graphs and statistical analyses were performed using the GraphPad Prism 10.1.2 program (GraphPad Software, Inc, La Jolla, CA, USA). Data are presented as the mean ± standard deviation (SD) of three independent experiments. Statistical analyses were performed using Student’s unpaired *t*-test. Significance was indicated by *p* < 0.05.

## Results

### Effect of CwlM depletion on mycobacterial growth and morphological characteristics

**Fig 1:**
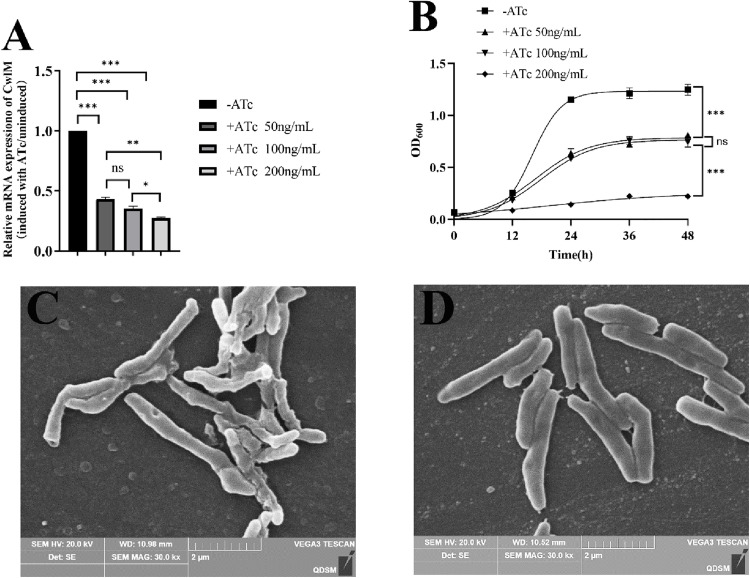
Effect of CwlM depletion on mycobacterial growth and morphological changes. (A) The CwlM-depleted strain of *M. smegmatis* was generated by CRISPRi technology. The graph displays the mean of the relative mRNA expression of CwlM normalized to sigA. (B) Growth curves of ATc-treated and untreated of *M. smegmatis*. (C–D) SEM images of *M. smegmatis* with (C) or without (D) ATc (magnification, × 30,000). The experiment was performed three times, and data are presented as the mean ± SD. Statistical significance is indicated as follows: *, p < 0.05, **, p < 0.01, ***, p < 0.001.

The depletion strain was constructed and validated by qRT-PCR. The results indicated that the transcription level of the CwlM gene was repressed to approximately 43.24% ± 1.25%, 35.36% ± 1.55%, and 27.27% ± 0.77% of that of the uninduced strain in the presence of ATc concentrations of 50, 100, and 200 ng/mL, respectively (Fig 1A). Notably significant CwlM depletion efficiency was observed at an ATc concentration of 200 ng/mL; however, under these conditions, the growth of the CwlM-depleted strain was markedly suppressed (Fig 1B). At ATc concentrations of 50 and 100 ng/mL, bacterial growth rates were reduced in comparison to that in uninduced bacteria, suggesting that CwlM depletion negatively affected *M. smegmatis* growth dynamics. No significant difference in growth rates was noted between induction with 50 and 100 ng/mL ATc. Consequently, subsequent experiments were conducted in the presence of 100 ng/mL ATc.

We performed SEM to assess the effects of CwlM on the cellular morphology of *M. smegmatis*. As illustrated in Fig 1C, the induced *M. smegmatis* strains treated with ATc displayed pronounced morphological alterations characterized by an irregular appearance featuring a wrinkled cell surface with indentations in the cell wall. By contrast, *M. smegmatis* in the absence of ATc exhibited a normal appearance, including a rod-like shape with a smooth cell surface. (Fig 1D).

### MIC determination of antibiotics

**Fig 2:**
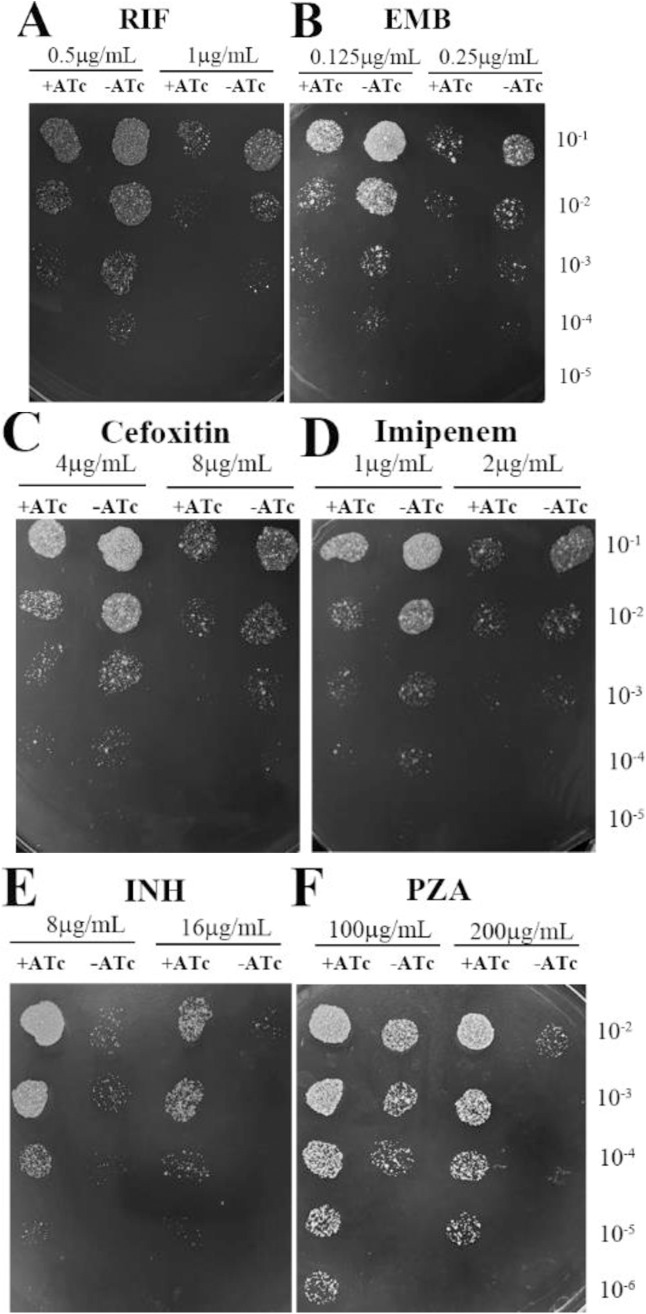
The growth of induced or uninduced *M. smegmatis* in the presence of different concentrations of anti-tuberculosis drugs. The cultures were diluted 10-fold and plated on Middlebrook 7H10 agar. The addition of ATc caused a one-log10 reduction in CFUs for the induced strain compared to uninduced bacteria after exposure to RIF (A), EMB (B), cefoxitin (C), and imipenem (D). Conversely, ATc led to a two-log10 increase in CFUs for the induced strains versus the control group following exposure to INH (E) and PZA (F).

To investigate the impact of CwlM depletion on mycobacterial sensitivity to anti-tuberculosis drugs, we assessed the MICs for four first-line anti-tuberculosis agents, namely INH, RIF, PZA, and EMB, as well as the β-lactams cefoxitin and imipenem. The induced strains exhibited increased susceptibility to RIF, EMB, cefoxitin, and imipenem, as indicated by 2-fold decreases in their MICs compared with those of their uninduced counterparts (Table 2). At both 0.5 × MIC and 1 × MIC concentrations of RIF, EMB, cefoxitin, and imipenem, the addition of ATc resulted in a one-log10 reduction in the number of colony-forming units (CFUs) for the induced strain compared with uninduced bacteria (Fig 2A–D).

**Table 2:** MICs of anti-tuberculosis drugs in the CwlM-depleted and control strains.

Strains	MIC(μg/mL)					
	INH*	RIF*	PZA*	EMB*	Imipenem*	Cefoxitin*
+ATc	16	0.5	200	0.125	1	4
-ATc	8	1	100	0.25	2	8

*, p < 0.05.

Unexpectedly, the induced strains displayed heightened tolerance to INH and PZA, as indicated by 2-fold increases in their MICs relative to uninduced strains (Table 2). The Alamar Blue assay further corroborated these findings, revealing similar two-fold increases in INH and PZA MICs for the induced strains relative to their uninduced counterparts (S1–S2 Figs). The introduction of ATc led to two-log10 increase in the number of CFUs for the induced strains compared with the control values following exposure to INH and PZA at both 1 × MIC and 2 × MIC concentrations (Fig 2E, F).

### EtBr uptake assay

**Fig 3:**
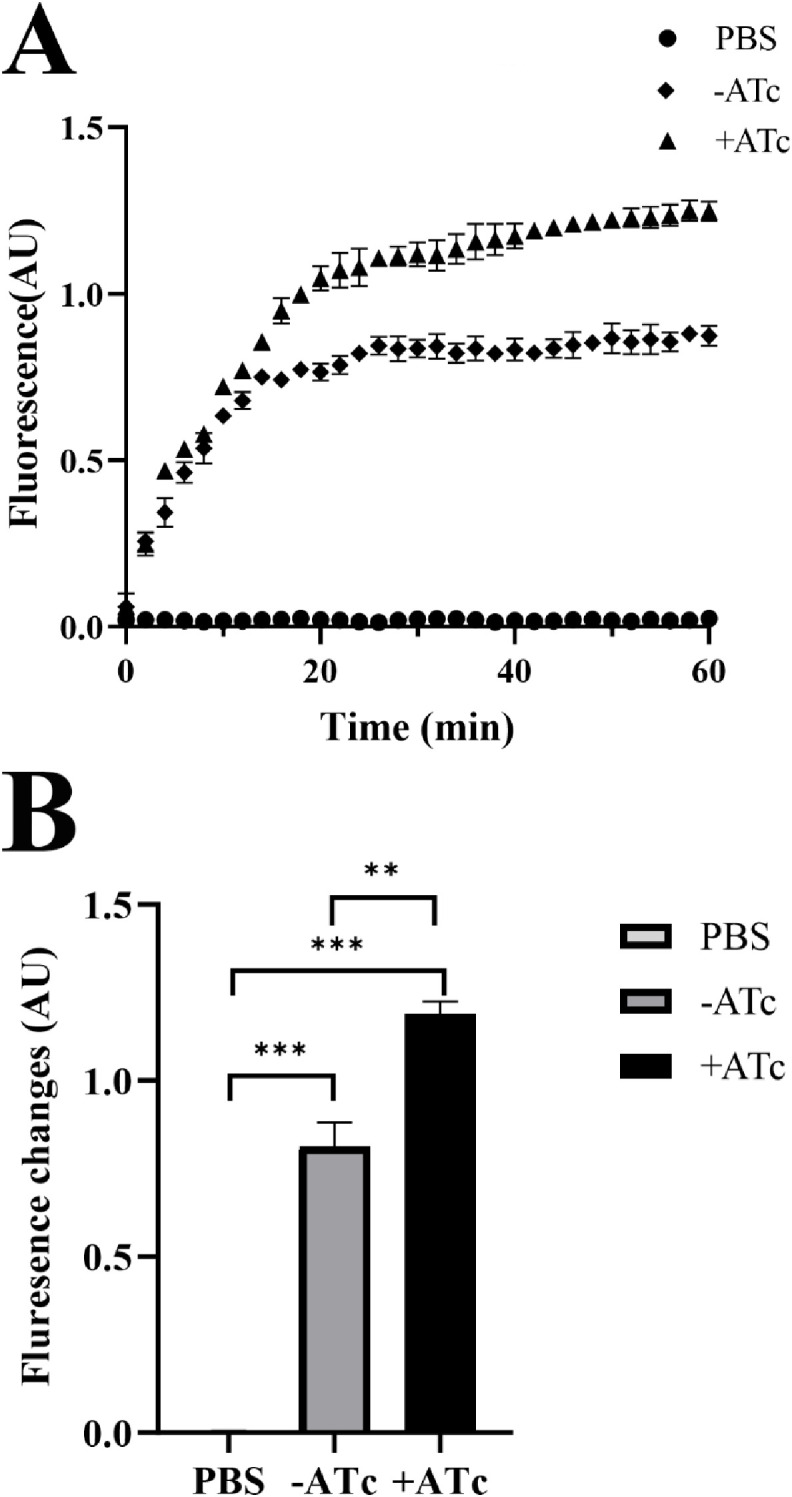
EtBr uptake assay. CwlM depletion resulted in increased EtBr uptake. (A) Induced and uninduced strains were incubated with 0.5 μg/mL EtBr, and fluorescence was monitored for 60 min. (B) Change in fluorescence was measured over the 60-min time course. PBS served as a blank control. The experiments were performed in triplicate and measured by fluorescence spectroscopy at 590 nm. Statistical significance is indicated as follows: **, p < 0.01, ***, p < 0.001.

To explore whether the alteration in antibiotic susceptibility was a secondary effect of cell permeability, we conducted an EtBr uptake assay across three independent experiments involving induced and uninduced strains. Our results indicated that the induced strain exhibited a significantly higher level of EtBr uptake than the control strain (Fig 3A,B). This increased EtBr uptake suggest enhanced permeability for anti-tuberculosis drugs, which could explain increased susceptibility to RIF, EMB, cefoxitin, and imipenem.

### DNA sequencing of KatG and PncA

To determine whether mutations in the KatG and PncA genes were responsible for tolerance to INH and PZA, we sequenced these genes. The alignment results revealed no mutations within either gene among the induced strains (S3–S4 Figs).

### Transcription levels of KatG, PncA, PzaA and LuxR-type regulator

**Fig 4:**
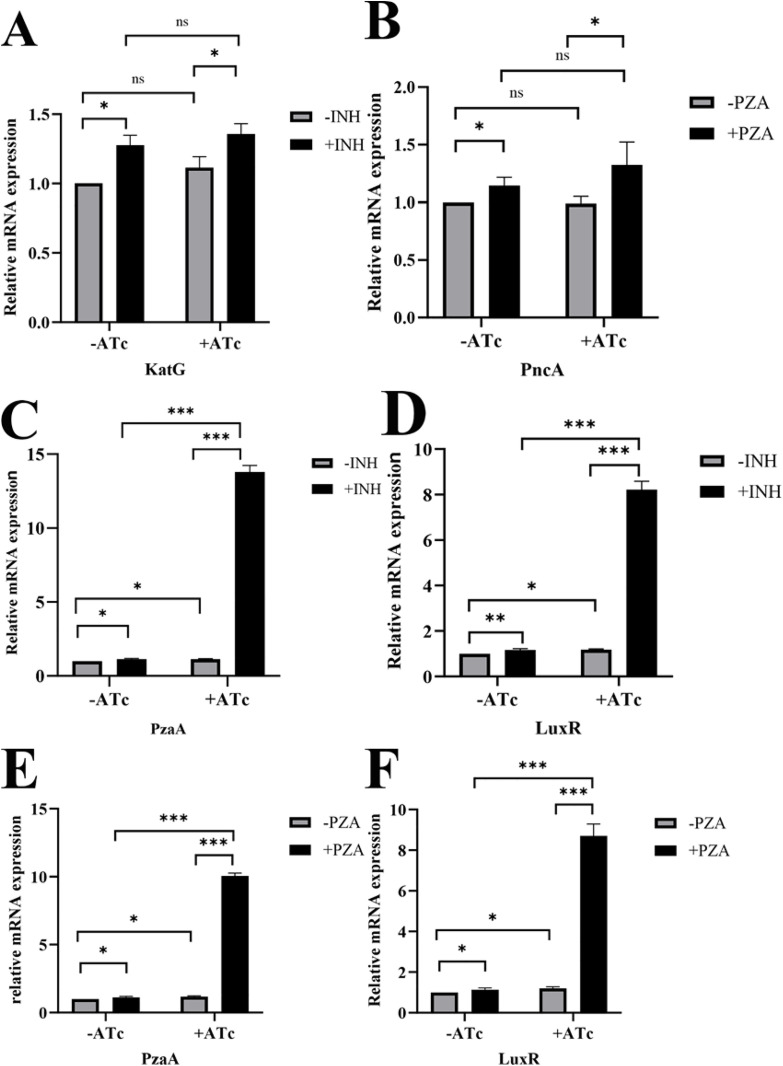
Effects of CwlM depletion on the transcription levels of KatG, PncA, PzaA, and LuxR-Type Regulator. (A-B) The transcription levels of KatG and PncA in induced and uninduced strains. (C-D) The transcription level of PzaA and LuxR with or without INH treatment in induced and uninduced strains. (E-F) In the presence or absence of PZA, the transcription levels of both PzaA and its LuxR-type regulator in induced and uninduced strains. Data are expressed as the mean mRNA level relative to that of the uninduced and INH-untreated strain, which was set to 1.0 for each gene (n = 3). Statistical significance is indicated as follows: *, p < 0.05, **, p < 0.01, ***, p < 0.001*.*

The qRT-PCR results indicated that the transcription level of KatG was significantly higher in the presence of INH than in its absence in both the induced and uninduced groups. However, no significant difference was found in the transcription level of KatG between induced and uninduced strains regardless of INH treatment (Fig 4A). More importantly, CwlM depletion does not affect KatG transcription. Similarly, when PZA was present, a marked increase in the transcription level of PncA was noted compared with that in conditions without PZA. Nevertheless, there was no significant difference in PncA transcription between induced and uninduced strains irrespective of PZA treatment (Fig 4B). These observations indicate that CwlM depletion does not influence the transcriptional activity of PncA. Thus, these findings imply limited involvement of KatG and PncA in tolerance to INH and PZA in induced strains.

Recently, a novel hydrazidase named PzaA has been identified in mycobacteria along with its LuxR-type regulator [7,9]. Our study revealed that the transcription levels for both PzaA (Fig 4C, D) and the LuxR-type regulator (Fig 4E,F) were significantly elevated in the induced group compared to those observed in uninduced bacteria, irrespective of the presence or absence of INH or PZA. This finding suggests that CwlM depletion enhances the transcriptional activity of both PzaA and its LuxR-type regulator. Such an enhancement may contribute to increased tolerance against INH. The transcription levels of both PzaA (Fig 4C, E) and its LuxR-type regulator (Fig 4D, F) were also higher in the presence of either INH or PZA than under conditions devoid of these agents.

### Identification and quantification of PzaA Protein

**Fig 5:**
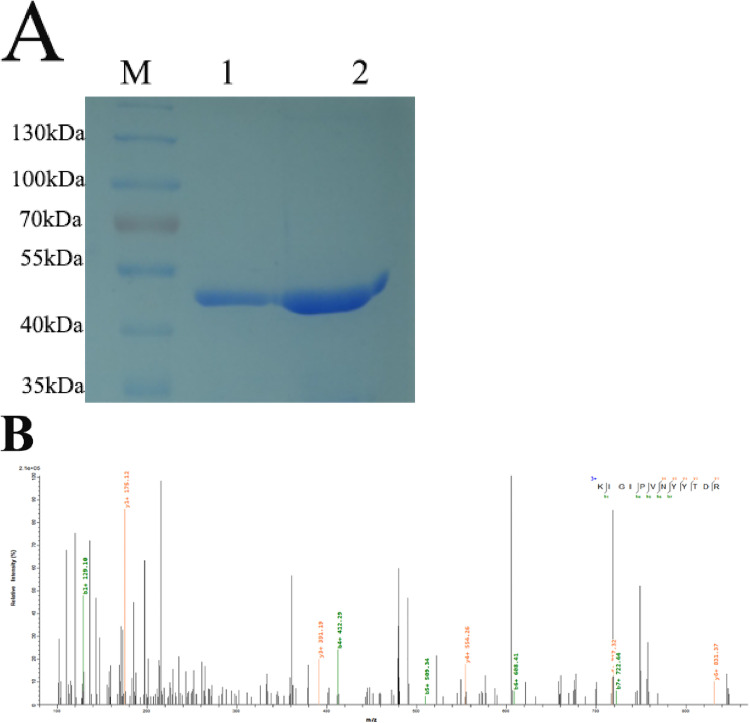
Purification and identification of PzaA protein from induced and uninduced strains. (A) SDS-PAGE analysis of purified proteins. M, PageRuler Prestained Protein Ladder (Thermo); 1, PzaA protein from uninduced strain; 2, PzaA protein from induced strain. (B) The unique peptide fragment of PzaA identified in MS analysis.

To further elucidate the distinct expression levels of PzaA between induced and uninduced strains, we isolated the PzaA protein from bacterial lysates. Utilizing SDS-PAGE, we identified a protein band with a molecular weight of 49 kDa, revealing a differential expression of this protein between the two strains. (Fig 5A). Mass spectrometry confirmed that the identified protein is PzaA (Fig 5B). The protein concentrations from the induced strain and uninduced strain were measured at 0.31 ± 0.05 mg/mL and 0.16 ± 0.02 mg/mL, respectively (p < 0.05). Therefore, the PzaA expression level in the induced strain was higher than that in the uninduced strain, which may account for its increased tolerance to INH.

### Confirmation of ΔPzaA strains and antibiotics susceptibility in ΔPzaA strain and ΔPzaA/CwlM-depleted strain

**Fig 6:**
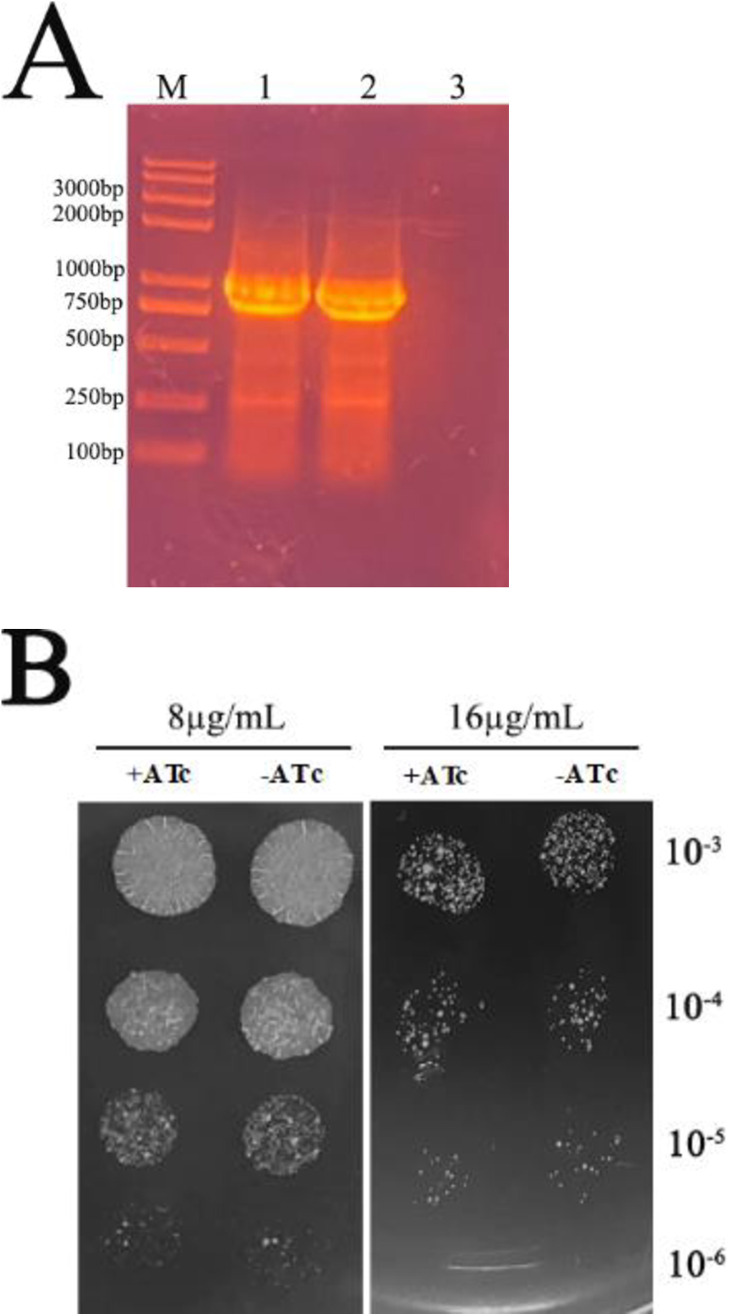
Confirmation of ΔPzaA strain and ΔPzaA/CwlM-depleted strain and antibiotics susceptibility of both strains. (A) Agarose gel electrophoresis of PCR products. M, Trans 2K Plus II DNA ladder (TRANS); lane 1-2, PCR products with an expected size (910 bp) from ΔPzaA strain and ΔPzaA/CwlM-depleted strain; lane 3, No PCR product from *M. smegmatis* mc^2^155 strain. (B) Antibiotics susceptibility assay. The graph shows there is no significant difference in CFUs between ΔPzaA strain and ΔPzaA/CwlM-depleted strain.

PCR analysis revealed an amplified product with the expected size of 910 bp (Fig 6A). Susceptibility assessments indicated that both ΔPzaA strain and ΔPzaA/CwlM-depleted strain exhibited equal sensitivity to INH (Fig 6B). This observation suggests that the tolerance to INH induced by CwlM depletion is potentially linked to elevated expression levels of PzaA.

### Transcriptomics analysis

**Fig 7:**
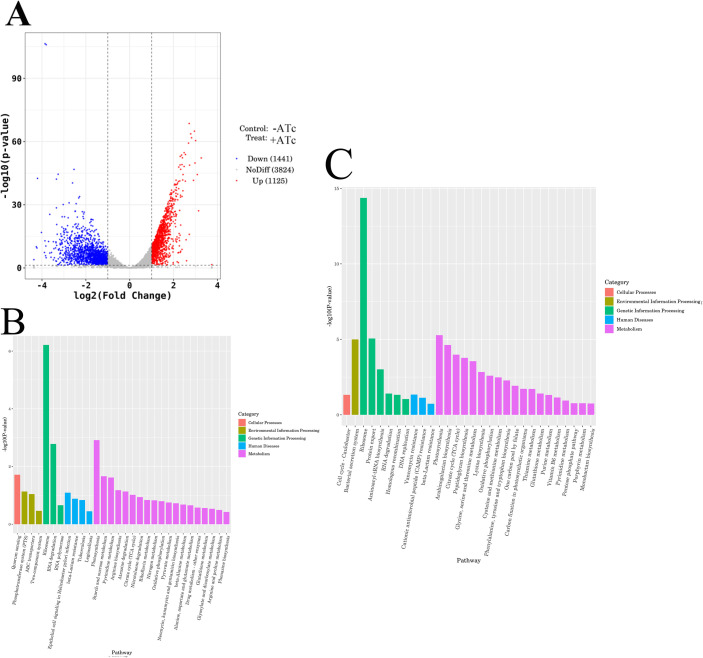
Transcriptomic analysis of induced and uninduced strains. (A) Volcano plot illustrating up-regulated and down-regulated transcripts in induced strains relative to uninduced strains. (B-C) KEGG analysis highlighting significantly up-regulated (B) and down-regulated (C) pathway genes in induced strains compared to uninduced strains. The results are shown as mean± SEM from 3 independent samples(n = 3) for each group.

We conducted a comparative analysis of transcriptional variations resulting from the depletion of CwlM (accession number CRA029376). The volcano plot revealed a distinct separation among transcriptional gene levels between induced and uninduced group (Fig 7A), indicating significant alterations in transcription profiles. KEGG enrichment analysis demonstrated that several pathways, including quorum sensing, ABC transporters, and other metabolic processes exhibited increased transcription levels in the induced group compared to those in the uninduced group (Fig 7B). Certain genes involved in peptidoglycan synthesis, as well as bacterial secretion systems and ribosomal function, showed decreased transcription levels (Fig 7C). Genes related to quorum sensing included LuxR-type regulators, other transcriptional regulators, and extracellular solute-binding proteins among others (S1 Table). The genes implicated in PG synthesis primarily comprised murA, murC, murD, murG, and pbpA (S2 Table), suggesting a potential impact of CwlM depletion on PG synthesis pathway.

## Discussion

In mycobacteria, CwlM plays a crucial role in regulating PG synthesis and cell wall remodeling [5,6]. This protein is believed to influence the antibiotic susceptibility of *M. abscessus* [7]. In this study, we employed the CRISPR technique to deplete CwlM to further investigate its characteristics. CwlM depletion resulted in slower growth rates and a wrinkled cell surface phenotype, indicating that the reduction of CwlM expression had a detrimental effect on *M. smegmatis* growth.

We subsequently assessed the drug susceptibility of the CwlM-depleted strain. We hypothesized that this strain might exhibit changes in susceptibility to anti-tuberculosis drugs. Surprisingly, the CwlM-depleted strain demonstrated heightened sensitivity to RIF, EMB, cefoxitin, and imipenem; conversely, it exhibited significantly greater tolerance to INH and PZA. According to EtBr uptake assays, this increased sensitivity might be attributable to enhanced permeability of the cell wall in the CwlM-depleted strain. It is essential to emphasize that, although *M. smegmatis* shares a similar cell wall architecture with *M. tuberculosis*, there are significant differences in the composition of lipids, polysaccharides and glycolipids between the two species. These variations result in distinct differences in bacterial growth, permeability, and drug sensitivity. Consequently, the effects of gene knockdown observed in *M. smegmatis* may not be directly applicable to *M. tuberculosis*. Further research is necessary to clarify the implications of these genetic modifications on *M. tuberculosis*.

Our study found that the observed tolerance to INH does not appear to be associated with gene mutations or variations in KatG expression. Similarly, tolerance to PZA was not associated with mutations or changes in PncA expression. These results suggest that alternative mechanisms underlie both INH and PZA tolerance. Recently, a novel amidase functioning as a hydrazidase was identified in mycobacteria [11,13]. This enzyme, designated PzaA (UniProtKB accession number: I7FF89), possesses the capability to degrade both INH and PZA, likely serving as a nutrient source for mycobacteria during periods of starvation [11]. This discovery also suggests its potential role as a tolerance factor against these drugs within mycobacterial species. Limited information has been reported regarding this enzyme. In *Microbacterium*, hydrazidase was noted to degrade acylhydrazides or paraben as their carbon sources [14,15]. Our study indicated that the heightened transcription of PzaA induced by CwlM depletion appears to contribute to the observed tolerance to INH. Additionally, we constructed ΔPzaA strain and ΔPzaA/CwlM-depleted strain; however, no significant difference in resistance to INH was revealed between these two strains. This observation further supports the hypothesis that the elevated expression of PzaA, also assessed through protein concentration measurements-induced by CwlM depletion, plays a role in conferring tolerance to INH. This represents the first report concerning PzaA’s involvement in INH tolerance. Although certain report has shown that PzaA is capable of degrading INH [11], there have been few reports on INH tolerance mediated by this enzyme. Thus, the conclusion that PzaA can mediate INH tolerance still demands further experimental investigations.

*M. smegmatis* is naturally resistant to pyrazinamide, mainly due to reduced uptake and increased efflux, which is different from *M. tuberculosis* [16–18]. The resistance of *M. tuberculosis* mainly depends on the conversion of pyrazinamide to pyrazinoic acid mediated by pncA. In this context, the observed resistance of CwlM-depleted strain to pyrazinamide in our study is more likely attributable to increased activity of efflux pumps. However, this phenomenon was not detected in our transcriptomic analysis. Further investigation is warranted to validate these findings in future studies.

It has been documented that the expression of PzaA can be induced by PZA, INH, and other analogs [11], consistent with our findings. Additionally, PzaA expression was found to be regulated by its LuxR-type regulator encoded by a gene (MSMEI_1050) located within the same gene cluster as PzaA [19]. In this context, we evaluated the transcription level of MSMEI_1050, finding that its transcription was significantly induced following CwlM depletion. LuxR-type transcriptional regulators are known to function as quorum-sensing regulators in bacteria [20]. They act as transcriptional activators responding to acyl-homoserine lactones or aromatic compounds [21,22]. In mycobacteria, some LuxR family genes could be regarded as putative cyclase proteins or as putative transcriptional regulators [23]. Clarifying the relationship between CwlM depletion and the elevated transcription levels of both PzaA and its associated LuxR-type regulator presents a complex challenge. In various bacteria, including *E. coli* [24], *Staphylococcus aureus* [25], and *Acinetobacter baumannii* [26,27], deletions or mutations in enzymes involved in PG synthesis and recycling have been found to result in alterations in drug susceptibility. The underlying mechanisms might involve these deletions or mutations, leading to either shortages or increases in specific metabolites that could act as signals inducing the expression of enzymes (or their regulators) associated with antibiotic tolerance or resistance [27,28]. Therefore, we hypothesize that CwlM depletion in mycobacteria may influence alternative products during PG synthesis and recycling processes. Transcriptomic analysis conducted in this study indicates that genes implicated in peptidoglycan synthesis—such as MurA, MurC, MurD, MurG, and pbpA—were downregulated. Notably, MurA is the first enzyme involved in peptidoglycan precursor synthesis and can be activated by phosphorylated CwlM [4]. This downregulation of MurA or other related genes could potentially alter the metabolites produced during PG synthesis, which might subsequently induce the expression of PzaA or LuxR-type regulators through an unknown mechanism.

It is noteworthy that the pathogenic strains such as *M. tuberculosi*s and *M. avium* lack an orthologous protein corresponding to PzaA [11]. Research has demonstrated that *M. tuberculosis* possesses N-acetyltransferases responsible for catalyzing the acetylation of INH [29,30,31]. Following this process, acetylisoniazid undergoes hydrolysis through an unspecified mechanism. It is speculated that PzaA participates in the hydrolysis of acetylisoniazid. In *Microbacterium* sp., hydrazidase has indeed been reported to hydrolyze acylhydrazides to their corresponding acids and hydrazine derivatives [14,32]; however, whether an amidase similar to PzaA is responsible for degrading INH or acetylisoniazid in pathogenic mycobacteria requires further identification.

## Supporting information

S1 Fig INH MIC by Alamar blue assay. Alamar Blue assay showed comparable two-fold increases in INH MIC for the induced strains relative to uninduced strains.
(TIF)

S2 Fig PZA MIC by Alamar blue assay. Alamar Blue assay showed comparable two-fold increases in PZA MIC for the induced strains relative to uninduced strains.
(TIF)

S3 Fig KatG alignment. The alignment results indicated that there were no mutations in the KatG gene among the induced strains.
(TIF)

S4 Fig PncA alignment. The alignment results indicated that there were no mutations in the PncA gene among the induced strains.
(TIF)

S1 Table Up-regulated pathways in induced group compared to uninduced group. (XLSX)

S2 Table Down-regulated pathways in induced group compared to uninduced group. (XLSX)
