# Supplementary figures and images for: The impact of CwlM depletion on the susceptibility of Mycobacterium smegmatis to anti-tuberculosis drugs
Source: PLoS One. 2025 Oct 24;20(10):e0334937. doi: 10.1371/journal.pone.0334937 (PMC12551912; doi:10.1371/journal.pone.0334937)

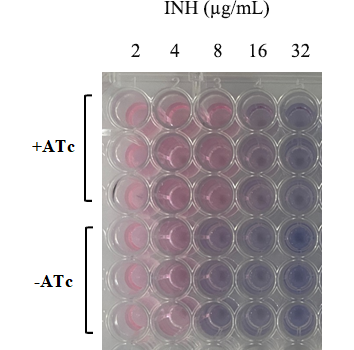

Supplement: S1 Fig — Alamar Blue assay showed comparable two-fold increases in INH MIC for the induced strains relative to uninduced strains. (TIF) [file pone.0334937.s001.tif]
